# Development of a Minimalistic Physiologically Based Pharmacokinetic (mPBPK) Model for the Preclinical Development of Spectinamide Antibiotics

**DOI:** 10.3390/pharmaceutics15061759

**Published:** 2023-06-17

**Authors:** Keyur R. Parmar, Pradeep B. Lukka, Santosh Wagh, Zaid H. Temrikar, Jiuyu Liu, Richard E. Lee, Miriam Braunstein, Anthony J. Hickey, Gregory T. Robertson, Mercedes Gonzalez-Juarrero, Andrea Edginton, Bernd Meibohm

**Affiliations:** 1 Department of Pharmaceutical Sciences, College of Pharmacy, University of Tennessee Health Science Center, Memphis, TN 38163, USA; kparmar@uthsc.edu (K.R.P.); pradeeplukka@gmail.com (P.B.L.); santosh.niper@gmail.com (S.W.); ztemrika@uthsc.edu (Z.H.T.); 2 Department of Chemical Biology, St. Jude Children’s Hospital, Memphis, TN 38105, USA; jiuyu.liu@stjude.org (J.L.); richard.lee@stjude.org (R.E.L.); 3 Department of Microbiology and Immunology, University of North Carolina, Chapel Hill, NC 27599, USA; miriam_braunstein@med.unc.edu; 4 Technology Advancement and Commercialization, RTI International, Durham, NC 27709, USA; ahickey@rti.org; 5 Mycobacteria Research Laboratories, Department of Microbiology, Immunology and Pathology, Colorado State University, Fort Collins, CO 80523, USA; gregory.robertson@colostate.edu (G.T.R.); mercedes.gonzalez-juarrero@colostate.edu (M.G.-J.); 6 School of Pharmacy, University of Waterloo, Waterloo, ON N2G 1C5, Canada; aedginto@uwaterloo.ca

**Keywords:** mPBPK, spectinamides, intratracheal PBPK, subcutaneous PBPK, interspecies extrapolation, granuloma model

## Abstract

Spectinamides 1599 and 1810 are lead spectinamide compounds currently under preclinical development to treat multidrug-resistant (MDR) and extensively drug-resistant (XDR) tuberculosis. These compounds have previously been tested at various combinations of dose level, dosing frequency, and route of administration in mouse models of *Mycobacterium tuberculosis* (*Mtb*) infection and in healthy animals. Physiologically based pharmacokinetic (PBPK) modeling allows the prediction of the pharmacokinetics of candidate drugs in organs/tissues of interest and extrapolation of their disposition across different species. Here, we have built, qualified, and refined a minimalistic PBPK model that can describe and predict the pharmacokinetics of spectinamides in various tissues, especially those relevant to *Mtb* infection. The model was expanded and qualified for multiple dose levels, dosing regimens, routes of administration, and various species. The model predictions in mice (healthy and infected) and rats were in reasonable agreement with experimental data, and all predicted AUCs in plasma and tissues met the two-fold acceptance criteria relative to observations. To further explore the distribution of spectinamide 1599 within granuloma substructures as encountered in tuberculosis, we utilized the Simcyp granuloma model combined with model predictions in our PBPK model. Simulation results suggest substantial exposure in all lesion substructures, with particularly high exposure in the rim area and macrophages. The developed model may be leveraged as an effective tool in identifying optimal dose levels and dosing regimens of spectinamides for further preclinical and clinical development.

## 1. Introduction

In 2018, the World Health Organization (WHO) reported that 10 million people developed tuberculosis (TB) and 1.5 million died of the disease. In the same year WHO reported 484,000 new cases of multidrug-resistant (MDR) TB worldwide, of which 6.2% were estimated to have extensively drug-resistant (XDR) TB [1]. In MDR TB, bacteria are at least resistant to the first-line anti-TB agents isoniazid and rifampin. XDR TB is a subtype of MDR TB that is additionally resistant to most of the other first- and second-line anti TB agents currently in use. With this high number in drug-resistant cases, the treatment of TB with the standard first- and second-line therapy is becoming increasingly difficult. Therefore, there is an urgent need for novel anti-TB drugs that assist in the management of drug-resistant TB, especially MDR or XDR TB infections [2,3]

Spectinamides are a novel class of anti-TB agents that are currently under preclinical development [4]. The lead compounds 1599 and 1810 are reported to have promising efficacy against *Mycobacterium tuberculosis* (*Mtb*) when tested in mouse models of TB infection at various dosage levels, dosage regimens, and routes of administration [2,5,6,7]. TB usually affects the lungs; however, it may also manifest systemically, in the spleen, and other organs. Therefore, it is imperative to understand the tissue distribution of any new anti-TB drug candidate.

In the last decade, physiologically based pharmacokinetic (PBPK) modeling and simulation has established a prominent role in the model-informed drug development paradigm impacting various stages of drug development, from early compound selection for first-in-human trials to dosing recommendations in product labeling [8]. PBPK models are mechanistic compartmental models that utilize the known anatomical and physiological knowledge base for humans and various preclinical species, in conjunction with the known physicochemical properties of a drug candidate to describe its in vivo concentration-time profiles in plasma and tissues after administration [9]. Each compartment in a whole-body PBPK model represents an organ or tissue of known volume, weight, and blood flow rate that when integrated with the physicochemical properties of the drug can holistically describe the drug disposition in each of these organs and tissues, and can thus provide a mechanistic understanding of drug disposition [10]. Consequently, the PBPK approach can be used to predict the pharmacokinetics of drug candidates in organs/tissues of interest solely based on the integrated species-specific physiological parameters and the drug-specific physicochemical properties, and can extrapolate their disposition behavior between different preclinical species and humans in support of drug development activities.

The objective of this study was to build, qualify, and refine a PBPK model for spectinamides as anti-tuberculosis drug candidates with the help of drug-specific parameters, and to observe plasma and tissue data in rodents (mouse and rat) after different routes of administration, including intravenous, subcutaneous, and intratracheal administration. This model is intended to describe and predict the pharmacokinetics of spectinamide compounds in various tissues, especially those relevant to *Mtb* infection, and across various species, thereby facilitating further drug development steps.

## 2. Materials and Methods

### 2.1. Chemicals and Reagents

Spectinamide 1599 (2-(5-chloroypyridin-2-yl)-N-((2R,4R,4aS,5aR,6S,7S,8R,9S,9aR, 10aS)-4a,7,9-trihydroxy-2-methyl-6,8-bis(methyl-amino)decahydro-2H-benzo[b]pyrano [2,3-e] [1,4]dioxin-4-yl)acetamide) and spectinamide 1810 (2-(5-hydroxypyridin-2-yl)-N-((2R,4R,4aS,5aR,6S,7S,8R,9S,9aR,10aS)-4a,7,9-trihydroxy-2-methyl-6,8-bis(methylamino) decahydro-2H-benzo[b]pyrano [2,3-e] [1,4] dioxin-4-yl)acetamide) were synthesized as previously described [11]. Spectinomycin purchased from Sigma-Aldrich (St. Louis, MO, USA) was used as an internal standard. Acetonitrile, methanol, HPLC grade water, formic acid, and nonafluoropentanoic acid were purchased from Fisher Scientific (Pittsburgh, PA, USA). Phosphate buffer saline (1× PBS) was purchased from Thermo Fisher Scientific (Waltham, MA, USA).

### 2.2. Animals

For studies in mice, six- to eight-week-old BALB/c mice were acquired from Jackson Laboratory (Bar Harbor, ME, USA) or Charles River (Wilmington, MA, USA). For studies in rats, 200 to 250 g Sprague-Dawley rats were obtained from Envigo (Indianapolis, IN, USA). Animals were kept on a 12 h light/dark cycle with access to food and water ad libitum, and were allowed to acclimatize for at least 3 days prior to any procedure. All animal experiments were conducted in accordance with the Animal Welfare Act and the Public Health Service Policy on Humane Care and Use of Laboratory Animals. Prior to initiation, all animal protocols were approved by the Institutional Animal Care and Use Committees of the University of Tennessee Health Science Center or Colorado State University, respectively.

### 2.3. Plasma Protein Binding Assay

Plasma protein binding was measured by equilibrium dialysis using a RED device (8 kDa molecular cutoff; Thermo Scientific, Rockford, IL, USA) containing plasma and buffer chambers for dialysis and a base plate. Two concentrations (0.5 and 5 mg/L) of 1599 and 1810 were prepared in mouse plasma from BALB/c mice and rat plasma from Sprague-Dawley rats, and an aliquot of 300 µL was added in the plasma chamber in duplicate. A 500 µL aliquot of PBS was added in the buffer chamber for dialysis. Then, the base plate was covered with sealing tape and incubated at 37 °C at approximately 100 rpm on an orbital shaker for 4 h to achieve equilibrium. After incubation, 50 µL of each sample was pipetted from the plasma and buffer chambers into separate micro-centrifuge tubes. A total of 50 µL of plasma was added to the buffer samples and an equal volume of PBS to the collected plasma samples and vortexed. Samples were processed by protein precipitation and analyzed for bound and unbound drug concentrations by liquid chromatography-tandem mass spectrometry (LC-MS/MS). Ranitidine (0.5 and 5 mg/L) was included as a positive control. The fraction of compound unbound to plasma proteins (*fu*) was calculated from the measured concentrations in the plasma and buffer chambers using the following equation:

(1) $$fu=1-\left(\frac{C(Plasma\;Chamber)-C(Buffer\;Chamber)}{C(Plasma\;Chamber)}\right)$$

### 2.4. Blood to Plasma Partition Ratio Assay

Fresh blood was collected from BALB/c mice and Sprague-Dawley rats via cardiac puncture in tubes with lithium heparin as anticoagulant. Hematocrit was measured using a VetScan HM5 hematology analyzer (Abaxis, Union City, CA, USA). For each species, control plasma was obtained from aliquots of the available blood by centrifugation at ~3750× *g* for 10 min at 4 °C. Aliquots of pre-warmed whole blood and plasma were spiked with the test compounds and incubated at 37 °C. After 1 h of incubation, the incubated whole blood was removed, and the plasma was separated by centrifugation. Aliquots of the incubated control plasma were also removed after 1 h of incubation. Plasma samples were treated with 8 volumes of ice-cold methanol containing internal standard (100 ng/mL spectinomycin) and analyzed for drug concentrations by LC-MS/MS [12].

The whole blood to plasma ratio *k*(*b*/*p*) was calculated by taking the ratio of the concentrations measured in the control plasma over the concentrations measured in the plasma centrifuged from the incubated whole blood, as shown in Equation (2). The erythrocyte to plasma partitioning coefficient *k*(*RBC*/*p*) was calculated from the whole blood to plasma ratio by accounting for the measured hematocrit, as shown in Equation (3):

(2) $$k(b/p)=\frac{C_{CP}}{C_{P}}$$

(3) $$k(RBC/p)=1+\left(\frac{1}{H}\right)\left[\left(\frac{C_{CP}}{C_{P}}\right)-1\right]$$

where *C_CP_* is the concentration of the test compound in the control plasma, *C_P_* is the concentration of the test compound in the plasma centrifuged from the incubated whole blood, and *H* is the measured hematocrit value.

### 2.5. Pharmacokinetic Studies in Healthy and Infected Mice

Single- and multiple-dose studies on the plasma and tissues pharmacokinetics of spectinamide compounds 1599 and 1810 in healthy mice after intravenous (IV), subcutaneous (SC), and intrapulmonary aerosol (IPA) administration were performed at the University of Tennessee Health Science Center. Pharmacokinetic (PK) assessments on infected animals were conducted in a dedicated biosafety level 3 (BSL-3) facility at Colorado State University. Details on these studies have been reported elsewhere and are summarized in Table 1 [5,7,13].

### 2.6. Pharmacokinetic Studies in Healthy Rats

Double catheterized (jugular and femoral vein) male and female Sprague–Dawley rats were used for single dose intravenous PK studies of spectinamide 1599 and 1810 with serial blood sampling to obtain the plasma concentration-time profiles in rats. Spectinamide 1599 or 1810 (10 mg/mL) was formulated in PlasmaLyte (Baxter International, Deerfield, IL, USA) and water (9:1) and injected via the femoral vein catheter as a rapid injection. For the serial blood sampling studies, six female and five male rats for spectinamide 1599 and six male rats (three separate studies) for spectinamide 1810 were dosed at 10 mg/mL (same formulation and administration as described above) and blood samples (250 µL) were collected via the jugular vein catheter at 0.08, 0.25, 0.5, 0.75, 1, 1.5, 2, 4, 6, 8, 10, 24, and 48 h after dosing. The plasma was separated from blood by centrifugation (~3750× *g* for 10 min at 4 °C) and stored at −70 °C until LC-MS/MS analysis. Urine samples were collected at predetermined time intervals (0–6, 6–10, 10–24, 24–48 h) up to 48 h after dosing, volumes were recorded, and samples were processed by protein precipitation and analyzed by LC-MS/MS. The fraction of drug excreted unchanged (*fe*) in urine at each time interval and a cumulative *fe* were calculated.

An additional single dose intravenous study of spectinamide 1599 with destructive sampling was performed to obtain the tissue PK profiles in rats. In this study, groups of four rats (two male and two female) were euthanized either at 15 min or at 4 h after dosing, and blood, lung, liver, spleen, and kidneys were collected. For tissue samples, the tissues were weighed and homogenized in four volumes of phosphate buffered saline (1× PBS) and stored at −70 °C until LC-MS/MS analysis.

### 2.7. Quantitative Analysis of Spectinamide Antibiotics

#### 2.7.1. Sample Preparation

Sample preparation was performed by protein precipitation using methanol. Plasma proteins were precipitated by the addition of 8 volumes (200 µL) of IS (spectinomycin; 100 ng/mL) in methanol to a volume of 25 µL of plasma/tissue homogenate test sample. Samples were vortexed for 30 s and centrifuged at 10,000× *g* for 10 min at 4 °C, and the supernatants were collected for LC-MS/MS analysis.

#### 2.7.2. Chromatographic Conditions

Chromatographic separations of the prepared samples were carried out using a Nexera XR liquid chromatograph (Shimadzu, Columbia, MD, USA) consisting of two pumps, online degasser, system controller, and auto sampler. A mobile phase consisting of (A) water with 5 mM ammonium formate buffer and (B) methanol with 5 mM ammonium formate buffer was used at a flow rate of 0.4 mL/min in gradient mode as follows: 0–1 min, 30% B; 1–2 min, 70% B; 2–4 min, 70% B; 4–5 min, 30% B, 5–7 min, 30% B. A HILIC^®^ 3.5 μm C_8_, 100 × 4.6 mm column (Phenomenex, Torrance, CA, USA) was used for the separation. Samples (5 µL) were injected on column and the eluate was led directly into the mass spectrometer.

#### 2.7.3. Mass Spectrometric Conditions

An API 4500 triple quadruple mass spectrometer (Applied Biosystems, Foster City, CA, USA) equipped with a turbospray ion source was operated in the positive ion mode. Selected reaction monitoring using precursor→product ion combinations of *m*/*z* 487.2→207.1, 418→207.1, and 365.1→333.2 was used for quantification of spectinamides 1599, 1810 and the IS spectinomycin, respectively.

### 2.8. Development of the PBPK Model

**Figure 1:**
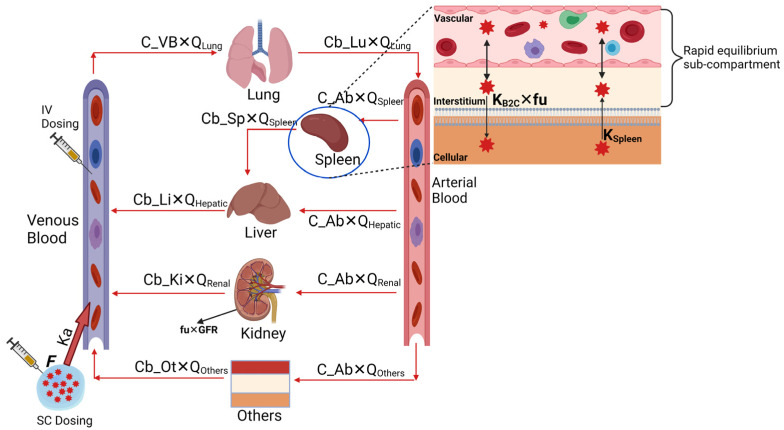
A schematic representation of the murine PBPK model accounting for intravenous and subcutaneous routes of administration. The model was established by estimating the key tissue distribution parameters by fitting the model to the observed plasma and tissue data and was subsequently qualified by simulating the concentration-time profiles at various dose levels and dosing regimens and comparing them to experimental data. The model is compartmentalized into blood (venous and arterial) and relevant tissues (lung, spleen, liver, kidney, and others), connected via blood flow rates (solid red arrows). Each tissue is divided into three sub-compartments, vascular, interstitial, and cellular. The vascular and interstitial sub-compartments were assumed to be in rapid equilibrium with blood, and the cellular sub-compartment was assumed to be in slow equilibrium with blood.

For the PBPK model base structure, we utilized, adapted, and expanded the work published by Nasu et al. [15]. The model structure included only the core tissues for which experimental data were available such as blood, lung, liver, spleen, and kidney, while all the remaining organs and tissues were lumped together for the mass balance of the compound. Thus, the developed model can be classified as a partial or minimal PBPK model. The base model consists of five tissues (lung, liver, spleen, kidney, and others) and blood compartments (venous and arterial) representing the actual anatomical structure. The tissues are connected in parallel between the arterial and the venous blood compartment. The lung receives the blood from the venous blood compartment via the pulmonary artery, and blood flows out into the arterial blood compartment via the pulmonary vein. All other tissues are supplied from the arterial blood compartment, and the blood leaving from these tissues, except for the spleen, flows directly into the venous blood compartment. The blood leaving the spleen flows into the liver. In this model, the unbound spectinamide 1599 and 1810 concentrations in plasma were assumed to be eliminated entirely by the kidney via glomerular filtration as suggested by our previous work [4]. Figure 1 represents the base model structure.

Each tissue is divided into three sub-compartments, vascular, interstitial, and cellular. The distribution between the vascular and interstitial sub-compartments was assumed to be instantaneous; thus, we have modeled both of these sub-compartments together. In contrast, the distribution between the interstitial and cellular sub-compartments was assumed to be slower and determined by first-order influx ($K_{I\to C}^{Organ}$) and back flux ($K_{C\to I}^{Organ}$) rate constants. We assumed that only the unbound fraction of spectinamide 1599 in plasma is distributed between the interstitial and cellular sub-compartments. Additionally, the concentration in plasma was calculated by the blood-to-plasma ratio, which was determined experimentally. All the physiological and physicochemical parameters used to build the PBPK model are listed in Table 2 and Table 3.

**Figure 2:**
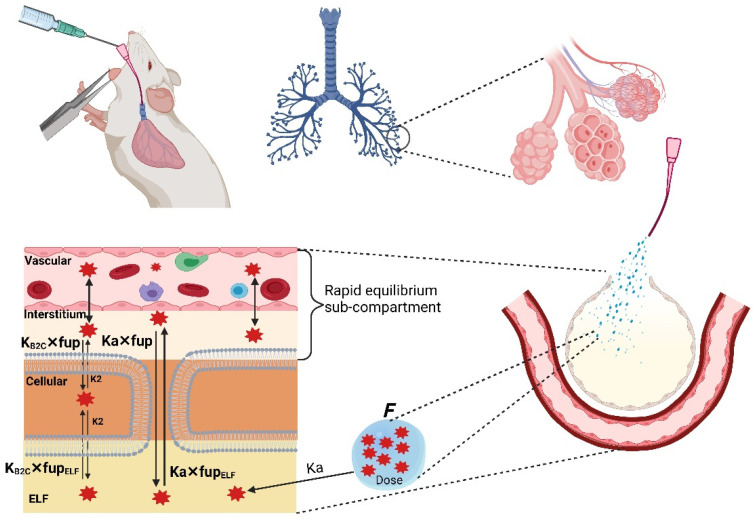
A schematic diagram of the modified lung compartment to model the drug dosing via intrapulmonary aerosol administration. The aerosol was modeled to be administered intratracheally, absorbed into the epithelial lining fluid (ELF) compartment, and distributed to the other sub-compartments of the lung, eventually reaching the systemic circulation.

For intravenous administration, the bolus dose was assumed to be mixed instantaneously into the venous blood. For subcutaneous administration, the dose was administered as an external compartment, absorbed into the venous compartment via a first-order absorption rate constant and a bioavailability component. For intrapulmonary aerosol administration, we have modified the lung tissue by adding the epithelial lining fluid (ELF) sub-compartment, as shown in Figure 2. The physiological volume of the ELF sub-compartment was obtained from the literature [18]. The dose was administered as an external compartment, absorbed into the ELF compartment via a first-order absorption rate constant and a bioavailability component. The compound was assumed to be bound to the ELF proteins, and only the unbound fraction was distributed to the other sub-compartments of the lungs and ultimately to the systemic circulation. The fraction unbound in ELF was calculated from the fraction unbound in plasma and the albumin ratio between plasma and ELF using the equation described by Poulin and Theil [19]. All the fixed and estimated parameters used to build the PBPK model are listed in Table 4.

We have used the same model structure for both spectinamides, 1599 and 1810. All modeling and simulation was performed in the Monolix Suite (Lixoft, Antony, France). The dynamic PK processes were described in terms of linear ordinary differential equations (ODE) written in MlxTran format, and are listed in the Supplementary Materials. All the model fittings were performed using the Stochastic Approximation Expectation Maximization algorithm of Monolix Version 2021R1 [20]. All plots were generated using ggplot2 in R [21].

### 2.9. Model Qualification

Qualification refers to how well the model fits or describes the observed data. We have utilized the commonly accepted model qualification criteria as described below [9]:

- Visual inspection of overlays of predicted and observed concentration-time profile indicating a reasonable agreement
- The observed data are within the 95% prediction interval of the model predictions
- The two-fold acceptance criteria between the observed and predicted exposures

For the intravenous model, the plasma and tissue (lung, liver, spleen, and kidney) PK data obtained after intravenous single dose (10 mg/kg) administration were used for model parameterization. The model qualification was performed utilizing the plasma and tissue PK data obtained after intravenous multiple dose (10 mg/kg daily dose for 5 days) administration. Similarly, the qualification of the subcutaneous and intrapulmonary model was performed with the plasma and tissue PK data obtained after the administration of single (10–200 mg/kg), twice-weekly (10–200 mg/kg), thrice-weekly (10–200 mg/kg), and daily dosing for 5 consecutive days (10–200 mg/kg).

### 2.10. Exploratory Simulation of Relative Drug Exposure in Granulomatous Lesion Substructures

A hallmark feature of pulmonary *Mtb* infection in humans is the formation of distinct caseous granuloma which may harbor a substantial number of bacilli [22]. One of the recurrent questions and challenges in anti-TB drug development is therefore to assess the exposure of new drug candidates not only in the interstitial sub-compartment of the lungs, but also in the substructures of granulomatous lesions [23]. While we did not have experimental data to build and implement granulomatous lesions into our murine PBPK model, we utilized the established multicompartmental human PBPK model of the lung and granuloma within the Simcyp simulator V21 R1 (Certara, Sheffield, UK) to translate our experimentally observed drug exposures in the lung interstitial sub-compartment into relative drug exposures in different granuloma substructures. This allowed us to gain an understanding of the relative relationships between exposures in different lesion compartments and the lung interstitial sub-compartment under the assumption that similar exposures in the lung interstitial sub-compartments as experimentally observed in our rodent studies could also be achieved in humans.

As described by Gaohua et al., the mechanistic multicompartment permeability-limited lung model in the Simcyp simulator consists of seven segments representing the upper and lower airways (two segments) and the lobes of the lung (five segments), and each segment is divided into four compartments (blood, mass, fluid, and air) [24]. This lung model has been extended to incorporate five additional compartments (capillary blood, interstitial fluid, macrophage, inner caseum, and outer caseum) representing the granuloma in the lung of patients with TB [25]. The drug-specific parameters utilized to define the compound (spectinamide 1599) within the Simcyp simulator were molecular weight (486.95), cLogP (−2.5184), pKa as diprotic base (8.69 and 6.95), *fu* and *k*(*b*/*p*) as listed in Table 2. The model calculated effective passive permeability (13.9 × 10^−4^ cm/s) in Simcyp was used to define the distribution of spectinamide 1599 within the granuloma compartments.

## 3. Results

### 3.1. PK Data for PBPK Model Development

The plasma and tissues PK data after single and multiple doses of spectinamide compounds 1599 and 1810 ranging from 10 to 200 mg/kg total daily dose in healthy mice after IV, SC, and IPA administration were generated at the University of Tennessee Health Science Center. An extensive set of dose-ranging and dose-fractionation studies for spectinamide 1599 and 1810 in the *Mtb* infected mice were performed at the Colorado State University, and the plasma PK data were used to check the predictive performance of our PBPK model. The plasma and tissue PK data after a single 10 mg/kg dose of spectinamide 1599 administered via intravenous route to healthy rats were also generated at the University of Tennessee Health Science Center. A comprehensive list of data sets utilized for the PBPK model development are summarized in Table 1.

### 3.2. Model Parameterization for Spectinamide 1599

The structure of our PBPK model was designed to include the tissues of interest in TB therapy such as blood (venous and arterial), lung, and spleen, as well as major elimination organs (liver and kidneys). All remaining tissues were lumped together as one compartment (called Other). During the model development process, we utilized three different approaches to obtain values for model-related parameters: (1) System-specific parameters (physiologic blood flow rates and organ volumes) were obtained from the literature. (2) Drug-specific parameters (*fu* and *k*(*b*/*p*)) were experimentally determined. (3) Transfer rate constants between model sub-compartments were estimated by fitting the model to experimentally observed data. Based on the observed biphasic disposition profiles of spectinamide compounds in plasma, lung, liver, spleen, and kidney after intravenous, subcutaneous, and intrapulmonary aerosol administration [2,5,6,7] and the known hydrophilic character of spectinamides 1599 and 1810 (cLogP −2.52 and −3.03, respectively), we assumed a model structure where each compartment consists of sub-compartments in rapid equilibrium with blood (vascular and interstitial) and a sub-compartment in slow equilibrium with blood (cellular). The model was parameterized in terms of first-order influx and back flux rate constants between the rapidly and the slowly equilibrating sub-compartments in each tissue. Thereby, the observed concentration-time profiles are the result of the distribution and elimination of the drug in various tissues.

### 3.3. Model Establishment for Intravenous Administration in Healthy Mice

**Figure 3:**
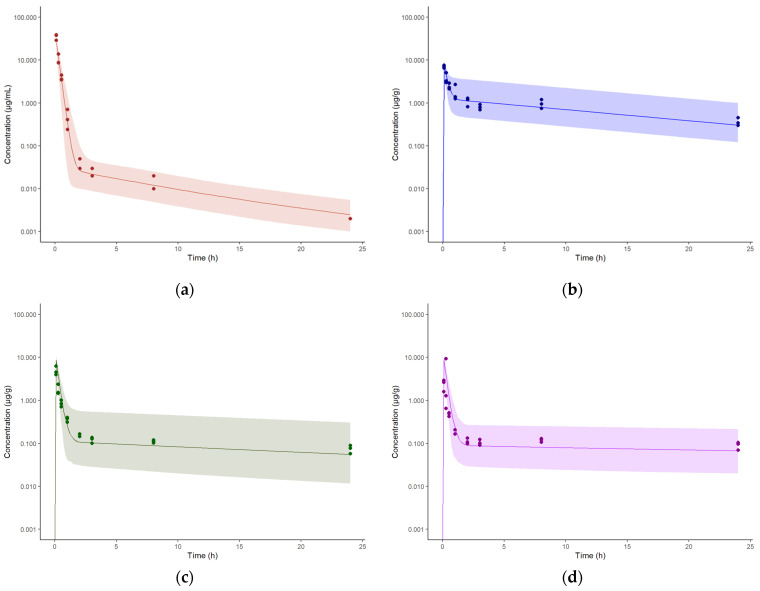
Concentration-time profiles of spectinamide 1599 in mouse plasma and tissues after administration of 10 mg/kg (IV): (**a**) Plasma (µg/mL); (**b**) Liver (µg/g); (**c**) Lung (µg/g); and (**d**) Spleen (µg/g). The solid line represents the simulated median concentration profiles, and the shaded region is the 95% prediction interval overlaid by the experimentally observed concentrations (*n* = 3 per sampling time point).

The physiological (blood flow rates and organ volumes) and the drug-specific parameters (*fu* and *k(b/p)*) were fixed and used as building blocks of the model, listed in Table 2 and Table 3. The remaining model parameters (tissue influx and back flux rate constants) were estimated by fitting the mouse PBPK model to the experimentally generated plasma, lung, liver, spleen, and kidney concentration-time data simultaneously, which were obtained after intravenous single dose administration of spectinamide 1599 (10 mg/kg). The parameters were assumed to have log-normal distribution and the residual unexplained variability (RUV) was characterized as a proportional error. The simulated median concentration-time profiles along with the 90% prediction intervals, overlaid by the observed concentrations, are shown in Figure 3.

### 3.4. Model Expansion to Intrapulmonary Aerosol Administration in Healthy Mice

**Figure 4:**
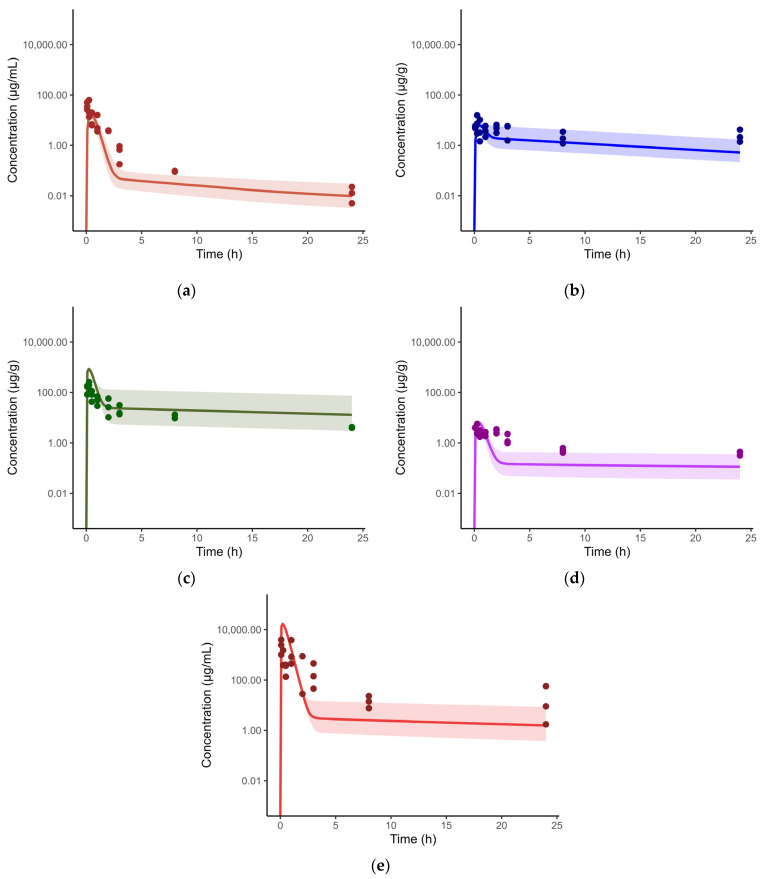
Concentration-time profiles of spectinamide 1599 in mouse plasma and tissues after administration of 50 mg/kg intrapulmonary aerosol administration: (**a**) Plasma (µg/mL); (**b**) Liver (µg/g); (**c**) Lung (µg/g); (**d**) Spleen (µg/g); and (**e**) ELF (µg/mL). The solid line represents the simulated median concentration profiles, and the shaded region is the 95% prediction interval overlaid by the experimentally observed concentrations (*n* = 3 per sampling time point).

**Figure 5:**
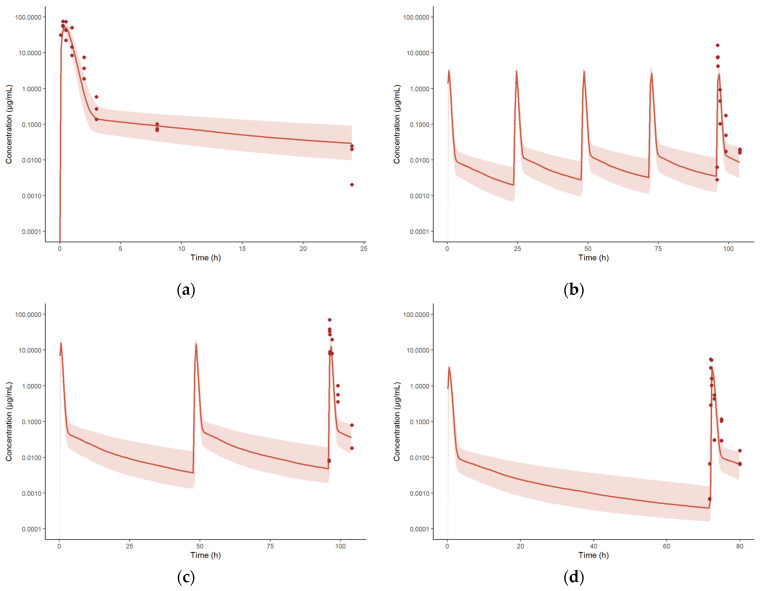
Concentration-time profiles of spectinamide 1599 in mouse plasma after single and multiple intrapulmonary aerosol administration at dose levels ranging from 10 to 150 mg/kg: (**a**) 150 mg/kg Single dose; (**b**) 10 mg/kg daily dosing for 5 consecutive days (QD5); (**c**) 50 mg/kg three times a week (TIW) dosing on Monday, Wednesday, and Friday; and (**d**) 10 mg/kg two times a week, dosing on Monday and Thursday. The solid line represents the simulated median concentration profiles, and the shaded region is the 95% prediction interval overlaid by the experimentally observed concentrations (*n* = 3 per sampling time point).

The developed intravenous model was extended to accommodate intrapulmonary aerosol (IPA) administration by including an external dosing compartment, from which spectinamide is absorbed into the ELF compartment via a first-order absorption rate constant (Ka) with a defined bioavailability (F). Figure 2 shows the schematic diagram of the modified lung compartment accommodating IPA administration. The Ka, F, and RUV were estimated by fitting the model to the observed plasma and tissue concentration-time profiles obtained after IPA single dose administration of spectinamide 1599 (50 mg/kg) in healthy mice. All tissue influx and back flux parameters were fixed to the values obtained from the intravenous model. The resulting model fits were overlaid with the experimental data and are shown in Figure 4. The model was used to simulate single (10 and 150 mg/kg) and multiple dose (QD5-once daily for 5 days, TIW-thrice weekly, and BIW-twice weekly at 10, 50, and 150 mg/kg dose levels) profiles. A select few plasma concentration profiles overlaid by observations are showed in Figure 5. The model was qualified by visual inspection of overlays of predicted and observed concentration-time profiles and the two-fold criteria between the predicted and simulated areas under the concentration-time curves (AUC).

### 3.5. Model Expansion to Subcutaneous Administration in Healthy Mice

**Figure 6:**
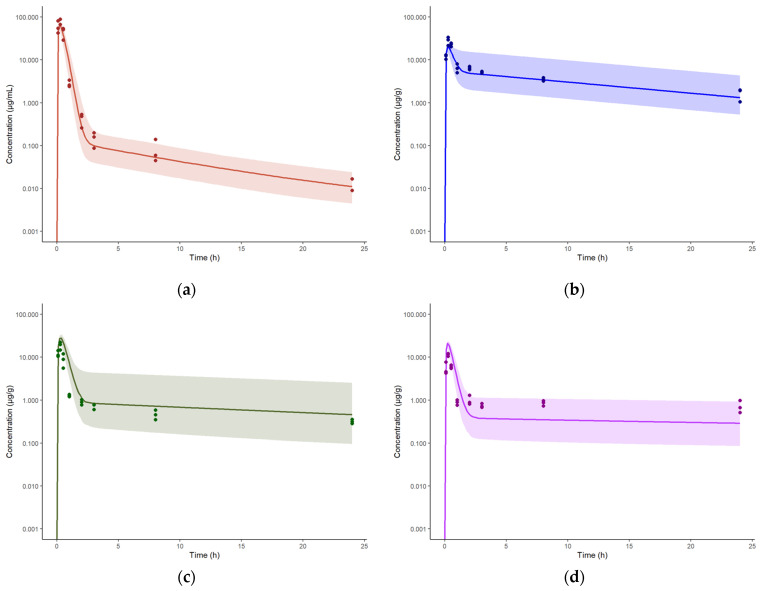
Concentration-time profiles of spectinamide 1599 in mouse plasma and tissues after subcutaneous administration of 50 mg/kg: (**a**) Plasma (µg/mL); (**b**) Liver (µg/g); (**c**) Lung (µg/g); and (**d**) Spleen (µg/g). The solid line represents the simulated median concentration profiles, and the shaded region is the 95% prediction interval overlaid by the experimentally observed concentrations (*n* = 3 per sampling time point).

Similar to IPA administration, the intravenous model was also extended to accommodate subcutaneous (SC) administration by including a SC dosing compartment from which spectinamide is absorbed into the venous blood compartment via a first-order absorption rate constant (Ka) and where dose is corrected for bioavailability (F). The Ka, F, and RUV were estimated by fitting the model to the observed plasma and tissue concentrations obtained after SC single dose administration of spectinamide 1599 (50 mg/kg) in healthy mice. Again, all tissue influx and back flux parameters were fixed to the values obtained from the intravenous model. The model was used to simulate single (200 mg/kg) and multiple dose (QD5, TIW, and BIW at 200 mg/kg) plasma and tissue concentration profiles. The model was qualified by visual inspection of overlays of predicted and observed concentration-time profiles as well as the two-fold criteria between the predicted and simulated AUCs (Figure 6).

**Figure 7:**
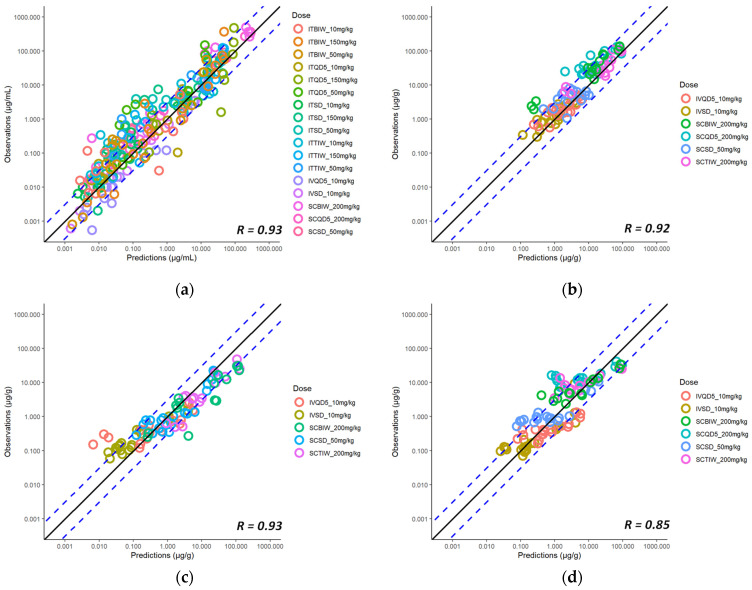
Predicted vs. observed plasma and tissue concentrations of spectinamide 1599: (**a**) Plasma (µg/mL); (**b**) Liver (µg/g); (**c**) Lung (µg/g); and (**d**) Spleen (µg/g) in healthy mice after intravenous (IV), subcutaneous (SC), and intrapulmonary aerosol (IPA) administration. SD-single dose; BIW-twice weekly on Monday and Thursday; QD5-once daily for 5 consecutive days (Monday to Friday); TIW-thrice weekly on Monday, Wednesday, and Friday. The solid black line represents the line of unity, dashed blue lines are the two-fold deviation, the symbols are the observed concentrations, and the R value is the Pearson correlation coefficient between the observations and predictions.

To describe the overall performance of the model with respect to the observations, Figure 7 shows the goodness-of-fit plots of observed vs. predicted concentrations for all the available plasma and tissue data from uninfected animals. The estimated model parameters for IV, SC, and IPA administration of spectinamide 1599 are listed in Table 4. Overall, the model predictions were in reasonable agreement with the observations, with less than a two-fold difference in the predicted and observed AUCs in plasma (Table 5) and tissues (Table 6).

### 3.6. Model Expansion to Infected Mice

**Figure 8:**
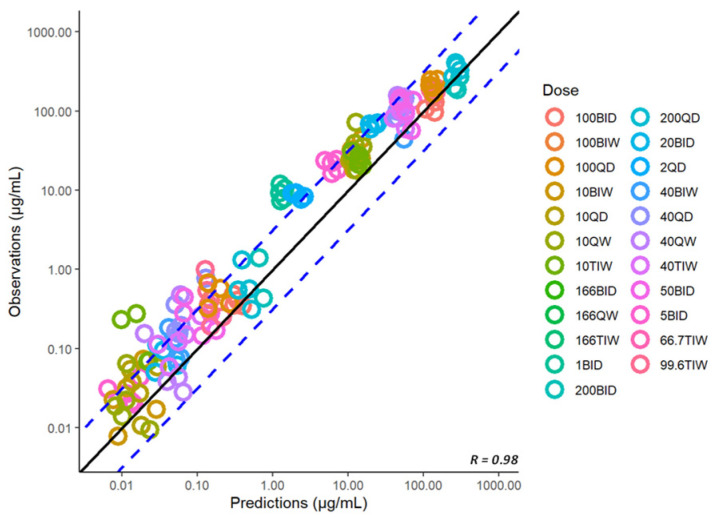
Predicted vs. observed plasma concentrations (µg/mL) of spectinamide 1599 in infected mice after different dosing regimens using subcutaneous administration. BID-twice daily; BIW-twice weekly; QD-once daily; QW-once weekly; TIW-thrice weekly. The solid black line represents the line of unity, dashed blue lines are the two-fold deviation, the symbols are the observed concentrations, and the R value is the Pearson correlation coefficient between the observations and predictions.

So far, we have utilized the model to characterize and predict the concentration-time profiles in healthy mice. Similarly, we have used the subcutaneous model to simulate the plasma concentration-time profiles in a set of dose-ranging and dose-fractionation studies that were performed in a standard mouse model of *Mtb* infection and were recently reported [5,7,13]. The model predictions were in reasonable agreement with the observations, as shown in the corresponding goodness-of-fit diagnostic plot of observed vs. predicted plasma concentrations in infected mice (Figure 8). Thus, the model developed for the healthy mice was able to predict the plasma concentration-time profiles in infected mice, suggesting no significant difference in the pharmacokinetics of spectinamide 1599 between healthy and infected mice. This finding is in agreement with our previously published work that also did not detect any differences in the pharmacokinetics of spectinamides between healthy mice and those infected with *Mtb* in chronic infection models [7].

### 3.7. Model Expansion to Rat as a Different Species

After the successful inter-route extrapolation of the model, we scaled the PBPK model between species by extending the model to rats. This was accomplished by incorporating the physiological parameters of a 225 g rat and updating the experimentally assessed drug-specific parameters *k*(*b*/*p*) and *fu*. Predicted profiles in rat plasma, lung, liver, and spleen were overlaid by the observations obtained after intravenous single dose administration of spectinamide 1599 (10 mg/kg) to healthy rats. While the plasma concentrations were in good agreement, the concentrations in the lung, liver, and spleen were slightly underpredicted compared to the observations. We believe this is most likely due to residual blood in the collected tissues since the tissues were not perfused prior to harvesting.

**Figure 9:**
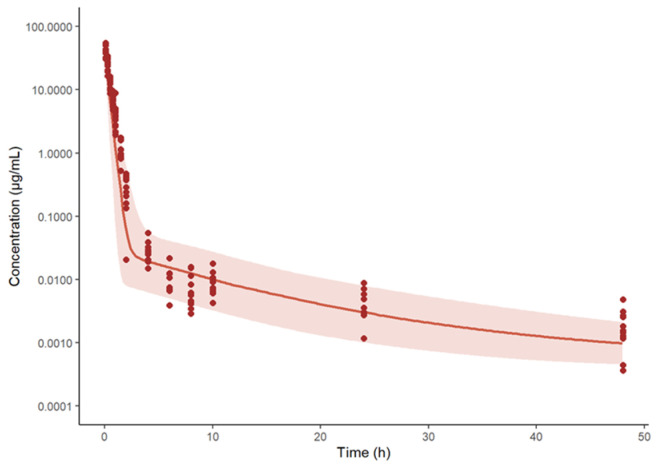
Concentration-time profiles of spectinamide 1599 in rat plasma after intravenous administration of 10 mg/kg. The solid line represents the simulated median concentration profiles, and the shaded region is the 95% prediction interval overlaid by the experimentally observed concentrations (*n* = 11 per sampling time point).

**Figure 10:**
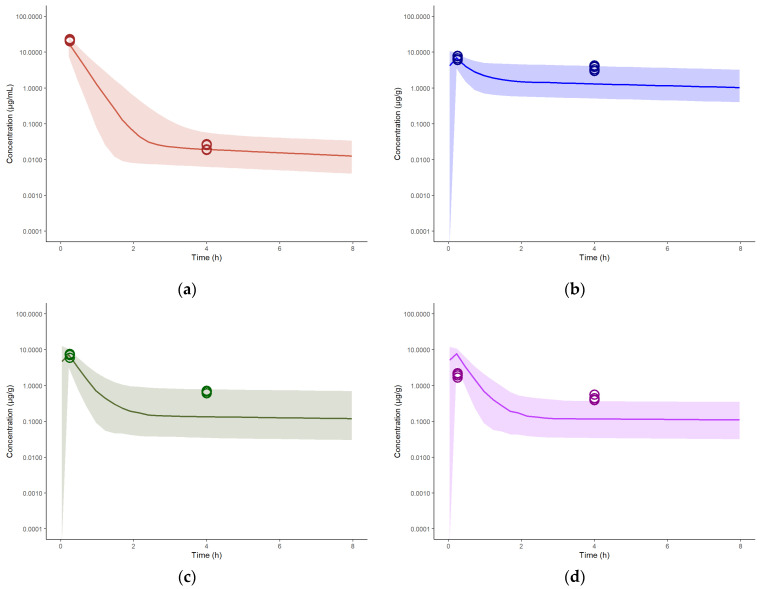
Concentration-time profiles of spectinamide 1599 in rat plasma and tissues after intravenous administration of 10 mg/kg: (**a**) Plasma (µg/mL); (**b**) Liver (µg/g); (**c**) Lung (µg/g); and (**d**) Spleen (µg/g). The solid line represents the simulated median concentration profiles, and the shaded region is the 95% prediction interval overlaid by the experimentally observed concentrations (*n* = 4 per sampling time point).

We have combined the mouse and rat model into a single integrated PBPK model for spectinamide 1599 that provides a single code structure that can estimate the parameters by fitting the model to the observed data in both species. We have also estimated the inter-individual variability on the influx rate constant of lung, liver, spleen, and ‘other tissue’ by fitting the model to the mouse and rat data (plasma and tissues) after intravenous single dose administration of spectinamide 1599. The inter-individual variability (ω^2^) was estimated as the variance ranging from 0.48 to 0.87, which corresponds to 50.9 to 106% CV in the tissue concentrations in rats and mice. The results are shown in Table 4, and the predicted profiles (median and predictive intervals) overlaid by the observation in plasma and tissues are shown in Figure 9 and Figure 10, respectively.

### 3.8. Model Expansion to Another Structurally Similar Compound, Spectinamide 1810

**Figure 11:**
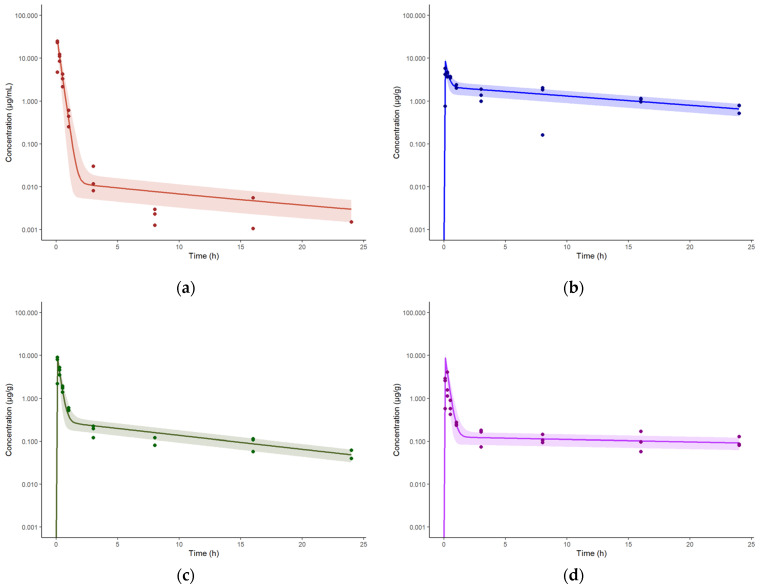
Concentration-time profiles of spectinamide 1810 in mouse plasma and tissues after intravenous administration of 10 mg/kg: (**a**) Plasma (µg/mL); (**b**) Liver (µg/g); (**c**) Lung (µg/g); and (**d**) Spleen (µg/g). The solid line represents the simulated median concentration profiles, and the shaded region is the 95% prediction interval overlaid by the experimentally observed concentrations (*n* = 3 per sampling time point).

**Figure 12:**
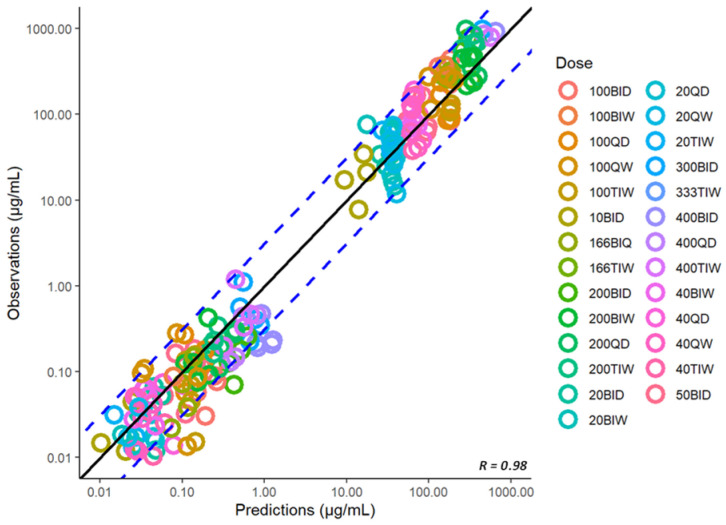
Predicted vs. observed plasma concentrations (µg/mL) of spectinamide 1810 in infected mice after different dosing regimens using subcutaneous administration. BID-twice daily; BIW-twice weekly; QD-once daily; QW-once weekly; TIW-thrice weekly. The solid black line represents the line of unity, dashed blue lines are the two-fold deviation, the symbols are the observed concentrations, and the R value is the Pearson correlation coefficient between the observations and predictions.

**Figure 13:**
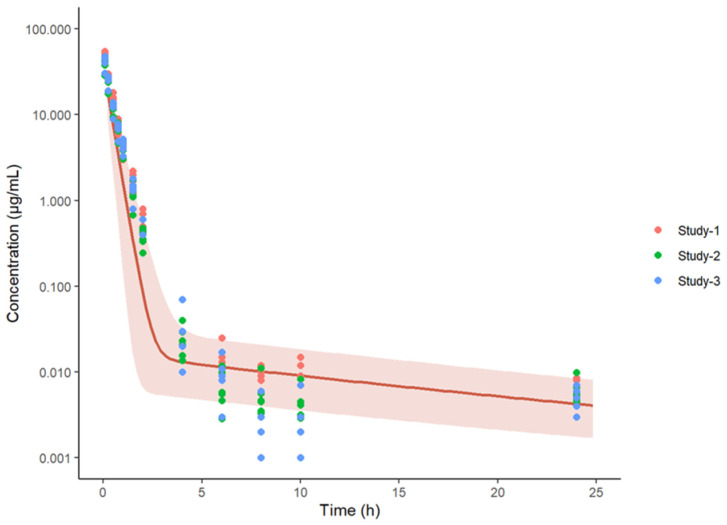
Concentration-time profiles of spectinamide 1810 in rat plasma after intravenous administration of 10 mg/kg. The solid line represents the simulated median concentration profiles, and the shaded region is the 95% prediction interval overlaid by the experimentally observed concentrations (*n* = 18 per sampling time point).

We expanded the established model to spectinamide 1810, a structurally highly similar spectinamide compared to 1599 by updating the drug-specific parameters in the model (*k*(*b*/*p*) and *fu*), and estimating ka, F, and residual error by comparing the model predictions with the plasma and tissue concentration-time PK profiles of spectinamide 1810 at various dose levels and dosing frequencies administered via the IV and the SC route in healthy mice. The estimated parameters are listed in Table 7. As indicated in Figure 11, predicted, and observed concentrations are in good agreement. The subcutaneous model was then used to also predict the 1810 plasma concentrations in infected mice, and the corresponding goodness-of-fit plot is shown in Figure 12. The model was scaled to rats to predict the plasma concentration-time profile after a single IV dose administration of spectinamide 1810 (10 mg/kg), and the corresponding predicted vs. observed data are plotted and shown in Figure 13. Overall, all model predictions for spectinamide 1810 were in reasonable agreement with the observations, with less than two-fold difference in the predicted and observed AUCs in plasma (Table 8) and tissues (Table 9).

Overall, these model expansions and qualification exercises across different dose levels and dosing frequencies, different routes of administration, different health status of the animals (infected vs. healthy), and different compounds within the same class established confidence in the predictive performance of the model in plasma and the specified tissues of interest.

### 3.9. Exploratory Assessment of Lesion Distribution of Spectinamide 1599 Based on the Simcyp Granuloma Model

We have utilized the multicompartment permeability-limited lung model with an active granuloma model within the Simcyp simulator to perform an exploratory assessment of relative exposures between the various granuloma substructures. The granuloma structure within the Simcyp simulator consisted of an outer rim of blood capillaries and interstitial fluid, enclosing a rim of macrophages and necrotic caseum (outer and inner). This was achieved by conducting simulations in the Simcyp simulator that achieved similar steady state concentrations (C_ss_) of spectinamide 1599 in plasma and the pulmonary blood reservoir (PBR) compartment of the Simcyp lung model similar to the observed C_ss_ in the plasma and the rapid equilibrium compartment of lung obtained following the efficacious subcutaneous dose of 200 mg/kg (QD5) in the standard mouse model of TB infection [5]. The drug-specific parameters utilized to define the compound (spectinamide 1599) within the Simcyp simulator are listed in Table S1 in the Supplementary Materials. The simulations were performed in a virtual healthy adult Caucasian population (*n* = 100). The virtual population received 6 mg/kg constant intravenous infusion (T_Infusion_ = 1 h) once a day for 5 consecutive days to achieve the similar C_ss_ as mentioned earlier. The corresponding Simcyp simulator files are also provided in the Supplementary Materials.

**Figure 14:**
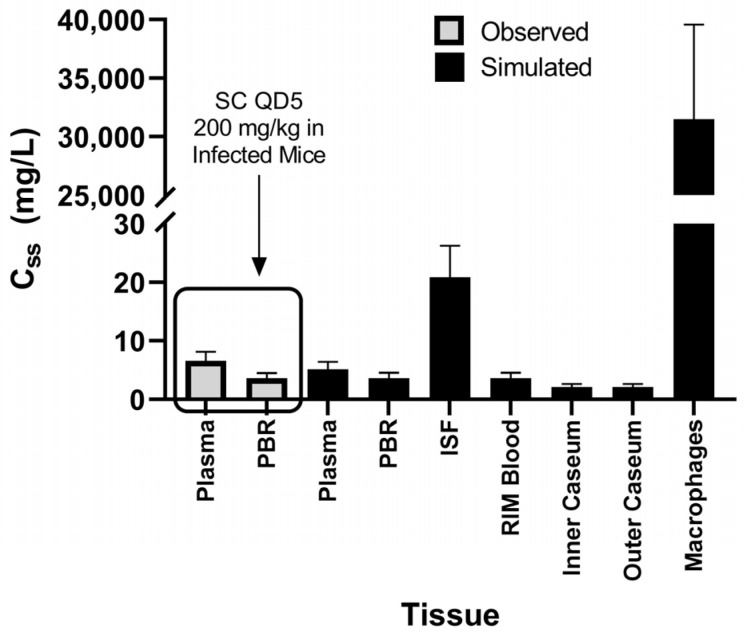
The observed and simulated steady state concentrations (mg/L) of spectinamide 1599 in plasma, pulmonary blood reservoir (PBR), rim-interstitial fluid, rim-capillary blood, macrophages, inner, and outer caseum. SC—subcutaneous, QD5—once daily for 5 consecutive days.

Figure 14 shows the comparative C_ss_ of spectinamide 1599 in plasma and pulmonary blood reservoir (observed and simulated). While the mean C_ss_ in the capillary blood of the granuloma was the same as the C_ss_ in PBR, the C_ss_ in rim-interstitial fluid (rim-ISF) was 5.7-fold higher than PBR. Additionally, the simulation showed a ~57% lower C_ss_ in the caseum of the granuloma compared to the PBR, while the C_ss_ in the macrophage was more than 8000-fold higher than PBR.

Spectinamide 1599 is reported to have a concentration-dependent intracellular uptake into murine-lung-derived dendritic cells and a BALB/c-derived monocyte macrophage cell line [26]. The simulated terminal half-life of spectinamide 1599 in the rim-macrophage (58 h) was 7.45-fold greater than the terminal half-life in the rim-blood (7.75 h). This slower elimination from the intracellular to the interstitial space, since the distribution is permeability limited, could explain the accumulation of spectinamide 1599 in the intracellular space, as reported for moxifloxacin [27]. The substantial accumulation in the macrophages suggests that spectinamide 1599 might be efficacious in clearing the intracellular bacilli.

## 4. Discussion

Characterizing the disposition of a compound in the tissues of interest is important in understanding the pharmacology of novel antibiotic agents. TB is primarily a pulmonary disease, but extrapulmonary manifestations have been reported in 10–42% of patients [28]. Thus, understanding the drug disposition and exposure in the tissues in which *Mtb* is residing is of utmost importance for any new antitubercular drug.

In this study, we have used PBPK modeling to integrate preclinical in vivo PK data for an investigational class of anti-tuberculosis compounds from numerous experiments comprising different dosing regimens, routes of administration, disease status, and species to characterize their disposition behavior. We chose a minimalistic PBPK modelling approach because of its three major advantages over empirical PK approaches: (1) richer information content: the model utilizes both drug-independent (organ volumes, blood flow rates, and other physiological parameters) and drug-specific (*fu*, *k*(*b*/*p*), permeability, clearance mechanism, etc.) parameters; (2) modular structure: the model compartmentalization is derived from the anatomical structure of the body which allows it to be adjusted for different conditions (e.g., disease status) and different routes of administration; and (3) universality: the PBPK model structure is common across all mammalian species, allowing it to scale the model from one species to another [29]. These features allowed the applicability of the PBPK model to understand the disposition of spectinamide compounds in plasma and tissues across various dosing levels, regimens, routes of administration, and species. Thus, the objective of this modeling exercise was to build a platform PBPK model which could be leveraged to predict the dose and regimens required to achieve desired drug exposures in target tissues relevant for TB infection in different preclinical species, and ultimately in humans.

As a first step, we compiled the physiological parameters relevant for the investigated species and integrated them with experimentally measured or calculated drug-specific parameters into a structural model. Both spectinamides considered, 1599 and 1810, exhibit low plasma protein binding in mouse and rat, and measured unbound percentages ranged from 56 to 70%. The measured blood to plasma ratio of 0.5 to 0.8 for both of the compounds in mouse and rat blood reflects a preferential distribution in plasma as compared to the blood cells. In the PBPK model, the blood-to-plasma ratio was used to convert the model predicted blood concentrations to plasma concentrations as determined in our experimental PK studies.

As a subsequent step, we decided on the model structure based on the available biphasic PK profiles in plasma and tissues for mice and rats [2,5,6,7]. The dynamic PK processes within the model compartments and sub compartments were described in terms of linear ordinary differential equations written in MlxTran format. The model parameters were estimated using a stochastic approximation expectation-maximization algorithm by fitting the model to the observed plasma and tissue data obtained after intravenous administration.

We have assumed a rapid equilibrium between the vascular and the interstitial or extracellular space of tissue by modeling them together. The distribution between the rapid equilibrium compartment and the cellular space was assumed to be permeability limited. The membrane permeation clearance is represented by the product of $K_{I\to C}^{Tissue}$, volume (interstitial and vascular), and fraction unbound in plasma. The estimated membrane permeation clearance values for all the tissues (lung, liver, spleen, kidney, other) were substantially lower than the organ blood flow rates (Q_T_), which supports our assumption of permeability-limited distribution.

One limitation of this approach is that these parameters are estimated by fitting the model to the data, making it more empirical than mechanistic. A more mechanistic approach would be to use the permeability surface area product as a membrane permeation clearance. However, we were unable to experimentally determine the passive permeability of spectinamides 1599 and 1810 with the parallel artificial membrane permeability assay (PAMPA) since spectinamides have very limited passive membrane permeability due to their high hydrophilicity and the concentrations in the acceptor compartment were below the limit of detection for the applied assay.

Therefore, we decided to estimate influx and efflux rate constants using the intravenous model fitting to the data obtained after single dose (10 mg/kg) administration, and subsequent qualification of the model by comparing model-based predictions to data obtained after an intravenous multiple dose (10 mg/kg QD5) study. Thereafter, the model was expanded to include the subcutaneous route of administration by incorporating a subcutaneous dosing compartment. The estimated first-order absorption half-life from the subcutaneous administration site for spectinamides 1599 and 1810 were ~10 and 5 min, respectively, and the bioavailability was 86 and 100%, respectively. The subcutaneous model was used to simulate an extensive set of dose-ranging and dose-fractionation studies for spectinamide 1599 and 1810 in *Mtb* infected mice [13]. A total of 23 dose groups for spectinamide 1599 ranging from 10 to 2000 mg/kg of total weekly dose were simulated and verified against the experimental observations. For spectinamide 1810, a total of 26 dose groups ranging from 20 to 4000 mg/kg of total weekly dose were simulated and verified against the observations. The model predictions were in reasonable agreement with the observations for both spectinamides, 1599 and 1810. This finding suggests that there is no significant difference in the pharmacokinetics of spectinamide 1599 and 1810 between healthy and infected animals, which is in agreement with our previously published work [7].

The intravenous model was also expanded to include the IPA route of administration by incorporating an external dosing compartment linked to the ELF compartment of the lung via a first-order absorption rate constant corrected for bioavailability. The estimated absorption half-life and bioavailability from the IPA administration site for spectinamide 1599 was ~8 min and 33%, respectively. The model was used to simulate single and multiple dose (QD5, TIW, and BIW) groups ranging from 10 to 150 mg/kg of total daily dose of spectinamide 1599 and verified against the observations. Overall, we have successfully utilized the developed PBPK model in performing inter-route and inter-drug extrapolation.

The inter-species extrapolation from mouse to rat was achieved by incorporating the physiological parameters of a healthy rat and updating the experimentally assessed drug-specific parameters (*fu* and *k*(*b*/*p*)), while fixing the distribution parameters (tissue influx and back flux) estimated from the intravenous mouse model. The model predicted concentration-time profiles in rat plasma were in reasonable agreement. However, the predicted concentrations in rat tissues (lung, liver, and spleen) were slightly underpredicted compared to the observations. This disagreement shows the variability in the tissue concentrations in mice and rats, which we hypothesized is due to the residual blood in the collected tissues, particularly in rats [30]. It would be interesting to test this hypothesis by performing a whole-body perfusion before harvesting the tissues, followed by measuring the concentration levels. However, this disagreement also exposed a potential limitation of our approach that is the reliance on one dataset at one dose level (10 mg/kg) used for the estimation of the model parameters, assuming dose-independent, linear pharmacokinetics of spectinamide 1599 and 1810. The latter, however, was reasonably well justified, as we had extensive PK data over a dose range of 10 to 400 mg/kg in mice that did not suggest any dose dependencies in disposition of these compounds [5,7].

Lastly, we performed an exploratory evaluation of relative steady state concentrations (C_ss_) of spectinamide 1599 in different granuloma compartments under the assumption that drug C_ss_ in plasma and the interstitial subcompartment of the lungs as observed in the standard mouse model of TB infection at a therapeutically efficacious dosing regimen could also be achieved in humans. The established granuloma model in the human Simcyp PBPK simulator was used to explore the disposition of spectinamide 1599 in granuloma compartments such as rim-interstitial fluid, macrophages where intracellular bacteria reside, and caseum (inner and outer) where bacteria with greatly reduced replication rate reside [25,31,32]. The simulations indicated that spectinamide 1599 achieves a substantially higher exposure in the rim-ISF compared to the C_ss_ in PBR and accumulates in the alveolar macrophages suggesting that spectinamide 1599 might be efficacious in clearing the intra and extracellular *Mtb*, while the lower C_ss_ in the inner and outer caseum could suggest still substantial but lower efficacy towards the non-replicating *Mtb* phenotypes. Lesion distribution studies of spectinamides in mouse models of chronic TB infection with advanced pulmonary lesions are ongoing to further explore these hypotheses.

## 5. Conclusions

The developed murine PBPK model was able to characterize and simultaneously predict plasma and tissue PK profiles of spectinamide 1599. The model successfully expanded and accounted for (a) various routes of administration including intravenous, subcutaneous, and intrapulmonary aerosol delivery; (b) healthy and infected animals; (c) different species, mice and rats; and d) another structurally similar compound, spectinamide 1810. The lesion distribution simulations, performed on Simcyp, showed higher steady state concentration (C_ss_) in the rim-interstitial fluid and alveolar macrophages than in plasma, suggesting good tissue penetration for potential efficacy in clearing diverse *Mtb* populations associated with human granulomas. The simulations also demonstrated a decent penetration of spectinamide 1599 in the necrotic caseum, suggesting substantial exposure in all lesion compartments. The overall modeling approach may be leveraged as an effective tool in planning and designing future development activities for spectinamide 1599 in higher species in the context of a model informed drug development (MIDD) paradigm [25,31,32,33].

## Figures and Tables

**Table 1:** Datasets for spectinamide 1599 and 1810 used for the PBPK modeling including administration routes, dose levels and dosing regimens, species, and disease status.

Drug	Species	Disease Status	Route of Administration	Dosing Frequency	Dose Level (mg/kg)	No. of Animals	No. of Data Points/Animal (Tissues)	No. of Animals/Sampling Time Point	Reference
Spectinamide 1599	BALB/c mice	Healthy	Intravenous	Single dose	10	24	1 (plasma, lung, liver, spleen)	3	[5]
Spectinamide 1599	BALB/c mice	Healthy	Intravenous	Daily dosing for 5 days (QD5)	10	21	1 (plasma, lung, liver, spleen)	3	[5]
Spectinamide 1599	BALB/c mice	Healthy	Subcutaneous	Single Dose	50	24	1 (plasma, lung, liver, spleen)	3	[5,6]
Spectinamide 1599	BALB/c mice	Healthy	Subcutaneous	Single Dose	200	27	1 (plasma, lung, liver, spleen)	3	[5,6]
Spectinamide 1599	BALB/c mice	Healthy	Subcutaneous	Daily dosing for 5 days (QD5)	200	18	1 (plasma, lung, liver, spleen)	3	[5,6]
Spectinamide 1599	BALB/c mice	Healthy	Subcutaneous	Twice a week (BIW)	200	18	1 (plasma, lung, liver, spleen)	3	[5,6]
Spectinamide 1599	BALB/c mice	Healthy	Subcutaneous	Three times a week (TIW)	200	18	1 (plasma, lung, liver, spleen)	3	[5,6]
Spectinamide 1599	BALB/c mice	Healthy	Subcutaneous	Three times a week (TIW)	200	18	1 (plasma, lung, liver, spleen)	3	[5,6]
Spectinamide 1599	BALB/c mice	Infected	Subcutaneous - Study 1A	Twice daily (QD5 for 4 weeks)	1, 5, 20, 50, 100, 200	30	2 (plasma)	5	[13]
Spectinamide 1599	BALB/c mice	Infected	Subcutaneous - Study 1A	Once daily (QD5 for 4 weeks)	2, 10, 40, 100, 200	25	2 (plasma)	5	[13]
Spectinamide 1599	BALB/c mice	Infected	Subcutaneous - Study 1A	TIW for 4 weeks	10, 40, 99.6	15	2 (plasma)	5	[13]
Spectinamide 1599	BALB/c mice	Infected	Subcutaneous - Study 1A	BIW for 4 weeks	10, 40	10	2 (plasma)	5	[13]
Spectinamide 1599	BALB/c mice	Infected	Subcutaneous - Study 1A	Once every week for 4 weeks	10, 40	10	2 (plasma)	5	[13]
Spectinamide 1599	BALB/c mice	Infected	Subcutaneous - Study 1B	Twice daily (QD5 for 4 weeks)	100, 166	12	2 (plasma)	6	[13]
Spectinamide 1599	BALB/c mice	Infected	Subcutaneous - Study 1B	TIW for 4 weeks	66, 166	12	2 (plasma)	6	[13]
Spectinamide 1599	BALB/c mice	Infected	Subcutaneous - Study 1B	BIW for 4 weeks	100	6	2 (plasma)	6	[13]
Spectinamide 1599	BALB/c mice	Infected	Subcutaneous - Study 1B	Once every week for 4 weeks	166	6	2 (plasma)	6	[13]
Spectinamide 1599	BALB/c mice	Infected	Subcutaneous - Study 1C	Twice daily (QD5 for 4 weeks)	50	6	2 (plasma)	6	[13]
Spectinamide 1599	BALB/c mice	Infected	Subcutaneous - Study 1C	Once daily (QD5 for 4 weeks)	100	6	2 (plasma)	6	[13]
Spectinamide 1599	BALB/c mice	Infected	Subcutaneous - Study 1C	TIW for 4 weeks	166	6	2 (plasma)	6	[13]
Spectinamide 1599	BALB/c mice	Infected	Subcutaneous - Study 1C	BIW for 4 weeks	100	6	2 (plasma)	6	[13]
Spectinamide 1599	BALB/c mice	Healthy	Intrapulmonary Aerosol	Single Dose	10, 50, 150	72	1 (plasma, lung, liver, spleen, ELF)	3	[5,6]
Spectinamide 1599	BALB/c mice	Healthy	Intrapulmonary Aerosol	QD5	10, 50, 150	54	1 (plasma, lung, liver, spleen, ELF)	3	[5,6]
Spectinamide 1599	BALB/c mice	Healthy	Intrapulmonary Aerosol	BIW	10, 50, 150	54	1 (plasma, lung, liver, spleen, ELF)	3	[5,6]
Spectinamide 1599	BALB/c mice	Healthy	Intrapulmonary Aerosol	TIW	10, 50, 150	54	1 (plasma, lung, liver, spleen, ELF)	3	[5,6]
Spectinamide 1599	Sprague-Dawley rats	Healthy	Intravenous	Single Dose	10	5 males/6 females	13 (plasma)	11	[4]
Spectinamide 1599	Sprague-Dawley rats	Healthy	Intravenous	Single Dose	10	4 males/4 females	1 (plasma, lung, liver, spleen)	4	Generated as described under Methods
Spectinamide 1810	BALB/c mice	Healthy	Intravenous	Single Dose	10	24	1 (plasma, lung, liver, spleen)	3	[7,13,14]
Spectinamide 1810	BALB/c mice	Healthy	Intravenous	QD5	10	24	1 (plasma, lung, liver, spleen)	3	[7,13,14]
Spectinamide 1810	BALB/c mice	Healthy	Subcutaneous	Single Dose	46	21	1 (plasma)	3	[7,13,14]
Spectinamide 1810	BALB/c mice	Healthy	Subcutaneous	Single Dose	50, 200	48	1 (plasma)	3	[7,13,14]
Spectinamide 1810	BALB/c mice	Healthy	Subcutaneous	QD5	50, 200	36	1 (plasma)	3	[7,13,14]
Spectinamide 1810	BALB/c mice	Infected	Subcutaneous - Study 2A	Twice daily (QD5 for 4 weeks)	10, 20, 50, 100, 200, 300, 500	35	2 (plasma)	5	[7,13,14]
Spectinamide 1810	BALB/c mice	Infected	Subcutaneous - Study 2A	Once daily (QD5 for 4 weeks)	20, 40, 100, 200, 400	25	2 (plasma)	5	[7,13,14]
Spectinamide 1810	BALB/c mice	Infected	Subcutaneous - Study 2A	TIW for 4 weeks	20, 40, 100, 200, 400	25	2 (plasma)	5	[7,13,14]
Spectinamide 1810	BALB/c mice	Infected	Subcutaneous - Study 2A	BIW for 4 weeks	20, 40, 100, 200	20	2 (plasma)	5	[7,13,14]
Spectinamide 1810	BALB/c mice	Infected	Subcutaneous - Study 2A	Once every week for 4 weeks	20, 40, 100	15	2 (plasma)	5	[7,13,14]
Spectinamide 1810	BALB/c mice	Infected	Subcutaneous - Study 2B	Twice daily (QD5 for 4 weeks)	50, 200	12	2 (plasma)	6	[7,13,14]
Spectinamide 1810	BALB/c mice	Infected	Subcutaneous - Study 2B	Once daily (QD5 for 4 weeks)	100	6	2 (plasma)	6	[7,13,14]
Spectinamide 1810	BALB/c mice	Infected	Subcutaneous - Study 2B	TIW for 4 weeks	166, 333	12	2 (plasma)	6	[7,13,14]
Spectinamide 1810	Sprague-Dawley rats	Healthy	Intravenous	Single Dose	10	18 males	13 (plasma)	18	Generated in this study as described under Methods

**Table 2:** Physiological and physicochemical parameters used to build the PBPK model for spectinamides 1599 and 1810.

Parameters	Values		Reference
	Mouse (20 g)	Rat (225 g)	
Q_Lung_ (L/h)	0.618	4.83	[16]
Q_Spleen_ (L/h)	0.00695	0.0412	[16]
Q_Liver_ (L/h)	0.139	0.901	[16]
Q_Kidney_ (L/h)	0.100	0.601	[16]
Q_Other_ (L/h)	0.371	3.29	[16]
GFR (L/h)	0.0168	0.088	[16]
V_Venous blood_ (L)	0.00120	0.0115	[17]
V_Arterial blood_ (L)	0.000515	0.00494	[17]
V_Lung_ (L)	0.000194	0.00140	[17]
V_Spleen_ (L)	0.000127	0.00277	[17]
V_Liver_ (L)	0.00193	0.0157	[17]
V_Kidney_ (L)	0.000525	0.00241	[17]
V_Other_ (L)	0.0235	0.245	[17]
V_ELF_ (L)	0.0000100	0.000100	[18]
Spectinamide 1599 *k*(*b*/*p*)	0.552	0.812	Generated in this study as described under Methods
Spectinamide 1599 *fu_Plasma_*	0.602	0.563	
Spectinamide 1599 *fu_ELF_*	0.948	0.940	
Spectinamide 1810 *k*(*b*/*p*)	0.604	0.785	
Spectinamide 1810 *fu_Plasma_*	0.693	0.607	
Spectinamide 1810 *fu_ELF_*	0.965	0.950	

Q represents blood flow; GFR represents glomerular filtration rate; V represents volume; ELF represents epithelial lining fluid; *k*(*b*/*p*) represents blood to plasma ratio; and *fu* represents fraction unbound.

**Table 3:** The volume fraction of tissue occupied by the vascular, interstitial, and cellular sub-compartments in mice and rats [17].

Tissues	Fraction Vascular	Fraction Interstitial	Fraction Cellular
Lung	0.26	0.19	0.55
Spleen	0.22	0.20	0.58
Liver	0.15	0.20	0.64
Kidney	0.10	0.15	0.75
Other	0.040	0.19	0.77

**Table 4:** Parameters, either fixed or estimated, used to build the PBPK model for spectinamide 1599.

Parameters	Description	Units	Intravenous Estimate (%RSE)	Intratracheal Estimate (%RSE)	Subcutaneous Estimate (%RSE)
$K_{I\to C}^{Lung}$	1st order uptake from the rapid equilibrium sub compartment (V+I) to the cellular sub compartment of the lung	1/h	0.068 (15.3)	Fixed	Fixed
$K_{C\to I}^{Lung}$	1st order back flux from the cellular sub compartment to the rapid equilibrium sub compartment of the lung	1/h	0.028 (41.5)	Fixed	Fixed
$K_{I\to C}^{Liver}$	1st order uptake from the rapid equilibrium sub compartment (V+I) to the cellular sub compartment of the liver	1/h	0.87 (10.1)	Fixed	Fixed
$K_{C\to I}^{Liver}$	1st order back flux from the cellular sub compartment to the rapid equilibrium sub compartment of the liver	1/h	0.061 (13.7)	Fixed	Fixed
$K_{I\to C}^{Spleen}$	1st order uptake from the rapid equilibrium sub compartment (V+I) to the cellular sub compartment of the spleen	1/h	0.048 (16.5)	Fixed	Fixed
$K_{C\to I}^{Spleen}$	1st order back flux from the cellular sub compartment to the rapid equilibrium sub compartment of the spleen	1/h	0.01 (106)	Fixed	Fixed
$K_{I\to C}^{Kidney}$	1st order uptake from the rapid equilibrium sub compartment (V+I) to the cellular sub compartment of the kidney	1/h	12.1 (19.7)	Fixed	Fixed
$K_{C\to I}^{Kidney}$	1st order back flux from the cellular sub compartment to the rapid equilibrium sub compartment of the kidney	1/h	0.15 (31.0)	Fixed	Fixed
$K_{I\to C}^{Others}$	1st order uptake from the rapid equilibrium sub compartment (V+I) to the cellular sub compartment of the other tissues	1/h	5.4 (4.92)	Fixed	Fixed
$K_{C\to I}^{others}$	1st order back flux from the cellular sub compartment to the rapid equilibrium sub compartment of the other tissues	1/h	7.0 × 10^−5^ (142)	Fixed	Fixed
*Ka*	1st order absorption rate constant	-	-	5.03 (4.53)	4.36 (7.86)
*F*	Bioavailability component	-	-	0.33 (2.03)	0.86 (6.15)
ω$K_{I\to C}^{Lung}$	$\mathrm{Inter}-\mathrm{animal}\;\mathrm{variability}\;\mathrm{on}\;K_{I\to C}^{Lung}$ estimated by simultaneously fitting the mouse and rat plasma and tissue data obtained after intravenous administration		0.87 (30.6)	Fixed	Fixed
ω$K_{I\to C}^{Liver}$	$\mathrm{Inter}-\mathrm{animal}\;\mathrm{variability}\;\mathrm{on}\;K_{I\to C}^{Liver}$ estimated by simultaneously fitting the mouse and rat plasma and tissue data obtained after intravenous administration		0.61 (29.8)	Fixed	Fixed
ω$K_{I\to C}^{Spleen}$	$\mathrm{Inter}-\mathrm{animal}\;\mathrm{variability}\;\mathrm{on}\;K_{I\to C}^{Spleen}$ estimated by simultaneously fitting the mouse and rat plasma and tissue data obtained after intravenous administration		0.66 (41.0)	Fixed	Fixed
ω$K_{I\to C}^{Other}$	$\mathrm{Inter}-\mathrm{animal}\;\mathrm{variability}\;\mathrm{on}\;K_{I\to C}^{Others}$ estimated by simultaneously fitting the mouse and rat plasma and tissue data obtained after intravenous administration		0.48 (21.6)	Fixed	Fixed
$\varepsilon_{Plasma}$	Proportional error for plasma concentration-time profile		0.32 (15.1)	6.57 (8.62)	0.54 (17.6)
$\varepsilon_{Lung}$	Proportional error for lung concentration-time profile		0.35 (14.4)	0.95 (9.92)	0.50 (15.3)
$\varepsilon_{Liver}$	Proportional error for liver concentration-time profile		0.28 (14.4)	2.55 (10.3)	0.29 (19.1)
$\varepsilon_{Spleen}$	Proportional error for spleen concentration-time profile		0.53 (14.4)	8.48 (9.86)	0.97 (16.5)

*K* represents first order rate constant; V+I represents the combined vascular and interstitial sub-compartments; ω is the inter-animal variance; and ε is the residual unexplained variance.

**Table 5:** Predicted vs. observed plasma exposure of spectinamide 1599 in healthy mice and rats.

Study	AUCinf (h × µg/mL)		
	Observed	Median Predicted	Fold Difference
IV SD 10 mg/kg	7.52	8.85	1.18
IV QD5 10 mg/kg	6.37	8.83	1.39
SC SD 50 mg/kg	40.4	39.8	1.02
SC QD5 200 mg/kg	227	159	1.43
IPA SD 10 mg/kg	5.51	4.87	1.13
IPA QD5 10 mg/kg	3.57	4.85	1.36
IPA TIW 10 mg/kg	5.36	4.83	1.11
IPA SD 50 mg/kg	23.2	24.4	1.05
IPA QD5 50 mg/kg	39.8	24.2	1.64
IPA TIW 50 mg/kg	24.3	24.2	1.00
IPA BIW 50 mg/kg	14.2	24.1	1.70
IPA SD 150 mg/kg	59.5	73.1	1.23
IPA QD5 150 mg/kg	96.9	72.7	1.33
IPA TIW 150 mg/kg	61.7	72.5	1.18
IPA BIW 150 mg/kg	108	72.4	1.49
Rat IV SD 10 mg/kg	19.8	12.5	1.58

IV-intravenous; SC-subcutaneous; IPA-intrapulmonary aerosol; SD-single dose; QD5-once daily for 5 consecutive days (Monday to Friday); TIW-thrice weekly (Monday, Wednesday, and Friday); BIW-twice weekly (Monday and Thursday).

**Table 6:** The predicted vs. observed exposure of spectinamide 1599 in lung, liver, and spleen.

Study	AUCinf (h × µg/g)								
	Lung			Liver			Spleen		
	Observed	Predicted	Fold Difference	Observed	Predicted	Fold Difference	Observed	Predicted	Fold Difference
IV SD 10 mg/kg	3.79	6.24	1.65	19.9	16.6	1.2	3.42	5.02	1.47
IV QD5 10 mg/kg	4.05	5.62	1.39	19.6	12.4	1.58	3.91	5.62	1.44
SC SD 50 mg/kg	19.1	27	1.41	91.8	70.5	1.3	23.6	21.2	1.11
SC QD5 200 mg/kg	69.1	97.9	1.42	336	217	1.55	110	96.4	1.14

IV-intravenous; SC-subcutaneous; SD-single dose; QD5-once daily dosing for 5 consecutive days (Monday to Friday).

**Table 7:** Parameters, either fixed or estimated, used to build the PBPK model for spectinamide 1810.

Parameters	Description	Units	Intravenous Estimate (%RSE)	Subcutaneous Estimate (%RSE)
$K_{I\to C}^{Lung}$	1st order uptake from the rapid equilibrium sub compartment (V+I) to the cellular sub compartment of the lung	1/h	0.13 (16.0)	Fixed
$K_{C\to I}^{Lung}$	1st order back flux from the cellular sub compartment to the rapid equilibrium sub compartment of the lung	1/h	0.076 (17.6)	Fixed
$K_{I\to C}^{Liver}$	1st order uptake from the rapid equilibrium sub compartment (V+I) to the cellular sub compartment of the liver	1/h	1.19 (12.0)	Fixed
$K_{C\to I}^{Liver}$	1st order back flux from the cellular sub compartment to the rapid equilibrium sub compartment of the liver	1/h	0.051 (17.2)	Fixed
$K_{I\to C}^{Spleen}$	1st order uptake from the rapid equilibrium sub compartment (V+I) to the cellular sub compartment of the spleen	1/h	0.059 (23.1)	Fixed
$K_{C\to I}^{Spleen}$	1st order back flux from the cellular sub compartment to the rapid equilibrium sub compartment of the spleen	1/h	0.013 (120)	Fixed
$K_{I\to C}^{Kidney}$	1st order uptake from the rapid equilibrium sub compartment (V+I) to the cellular sub compartment of the kidney	1/h	3.94 (43.9)	Fixed
$K_{C\to I}^{Kidney}$	1st order back flux from the cellular sub compartment to the rapid equilibrium sub compartment of the kidney	1/h	0.097 (45.1)	Fixed
$K_{I\to C}^{Others}$	1st order uptake from the rapid equilibrium sub compartment (V+I) to the cellular sub compartment of the other tissues	1/h	4.77 (7.88)	Fixed
$K_{C\to I}^{others}$	1st order back flux from the cellular sub compartment to the rapid equilibrium sub compartment of the other tissues	1/h	7.0 × 10^−5^ (0.00278)	Fixed
*Ka*	1st order absorption rate constant	-	-	8.26 (14.5)
*F*	Bioavailability component	-	-	1.00 (0.463)
ω$K_{I\to C}^{Other}$	Inter-animal variability on $K_{I\to C}^{Others}$ estimated by simultaneously fitting the mouse and rat plasma and tissue data obtained after intravenous administration		0.31 (14.7)	Fixed
$\varepsilon_{Plasma}$	Proportional error for plasma concentration-time profile		0.43 (14.7)	0.36 (15.6)
$\varepsilon_{Lung}$	Proportional error for lung concentration-time profile		0.13 (16.0)	0.30 (14.7)
$\varepsilon_{Liver}$	Proportional error for liver concentration-time profile		0.076 (17.6)	0.31 (14.7)
$\varepsilon_{Spleen}$	Proportional error for spleen concentration-time profile		1.19 (12.0)	0.43 (14.7)

*K* represents first order rate constant; V+I represents the combined vascular and interstitial sub-compartments; ω is the inter-animal variance; and ε is the residual unexplained variance.

**Table 8:** Predicted vs. observed plasma exposure of spectinamide 1810 in healthy mice and rats.

Study	AUCinf (h × µg/mL)		
	Observed	Median Predicted	Fold Difference
IV SD 10 mg/kg	7.91	7.64	1.04
IV QD5 10 mg/kg	9.45	7.70	1.23
SC SD 46 mg/kg	38.6	36.8	1.05
SC SD 50 mg/kg	67.9	40.0	1.70
SC SD 200 mg/kg	267	160	1.67
SC QD5 50 mg/kg	65.5	40.3	1.63
SC QD5 200 mg/kg	265	161	1.65
Rat IV SD 10 mg/kg	20.8	12.9	1.61

IV-intravenous; SC-subcutaneous; SD-single dose; QD5-once daily for 5 consecutive days (Monday to Friday).

**Table 9:** The predicted vs. observed exposure of spectinamide 1810 in lung, liver, and spleen.

Study	AUCinf (h × µg/g)								
	Lung			Liver			Spleen		
	Observed	Predicted	Fold Difference	Observed	Predicted	Fold Difference	Observed	Predicted	Fold Difference
IV SD 10 mg/kg	5.75	8.53	1.48	31.6	25.2	1.25	4.29	4.43	1.03
IV QD5 10 mg/kg	11.2	9.79	1.14	51.2	37.4	1.37	9.45	7.91	1.19

IV-intravenous; SD-single dose; QD5-once daily dosing for 5 consecutive days (Monday to Friday).

## Data Availability

Data generated in this study are available on request from the corresponding author.

## Supplementary Materials

The following supporting information can be downloaded at: https://www.mdpi.com/article/10.3390/pharmaceutics15061759/s1, Table S1: Model input parameters for the model development of spectinamide 1599 within the Simcyp simulator.
