# Supplementary material for: Development of a Minimalistic Physiologically Based Pharmacokinetic (mPBPK) Model for the Preclinical Development of Spectinamide Antibiotics
Source: Pharmaceutics. 2023 Jun 17;15(6):1759. doi: 10.3390/pharmaceutics15061759 (PMC10305115; doi:10.3390/pharmaceutics15061759)
Supplement: Supplementary file 1 [file pharmaceutics-15-01759-s001.zip › pharmaceutics-2360762-supplementary.pdf]

Pharmaceutics

Supplementary Materials: Development of a Minimalistic Physiologically-Based Pharmacokinetic (mPBPK) Model for the Preclinical Development of Spectinamide Antibiotics

Keyur R. Parmar, Pradeep B. Lukka, Santosh Wagh, Zaid H. Temrikar, Jiuyu Liu, Richard E. Lee, Miriam Braunstein, Anthony J. Hickey, Gregory T. Robertson, Mercedes Gonzalez-Juarrero, Andrea Edginton and Bernd Meibohm

Linear ordinary differential equations used to describe spectinamide concentrations in the different components of the developed minimalistic physiologically-based pharmacokinetic model:

Venous blood compartment

$$V^{Venous\ Blood} \times \frac{d}{dt} C^{Venous\ Blood} = Q^{Liver} \times Cb^{Liver} + Q^{Kidney} \times Cb^{Kidney} + Q^{Other} \times Cb^{Other} - Q^{Lung} \times Cb^{Lung} \quad \text{(S1)}$$

Venous plasma

$$C^{Venous\ Plasma} = \frac{C^{Venous\ Blood}}{k(b/p)} \quad \text{(S2)}$$

Lung compartment

Lung rapid equilibrium sub-compartment

$$\left(V_{Vascular}^{Lung} + \left(\frac{V_{Interstitial}^{Lung}}{k(b/p)}\right)\right) \times \frac{d}{dt} Cb^{Lung} = Q_{Lung} \times (C^{Venous\ Blood} - Cb^{Lung}) - K_{I\to C}^{Lung} \times fu \times Cb^{Lung} \times \left(V_{Vascular}^{Lung} + \left(\frac{V_{Interstitial}^{Lung}}{k(b/p)}\right)\right) + K_{C\to I}^{Lung} \times C_{Cellular}^{Lung} \times V_{Cellular}^{Lung} \quad \text{(S3)}$$

Lung cellular sub-compartment

$$V_{Cellular}^{Lung} \times \frac{d}{dt} C_{Cellular}^{Lung} = K_{I\to C}^{Lung} \times fu \times Cb^{Lung} \times \left(V_{Vascular}^{Lung} + \left(\frac{V_{Interstitial}^{Lung}}{k(b/p)}\right)\right) - K_{C\to I}^{Lung} \times C_{Cellular}^{Lung} \times V_{Cellular}^{Lung} \quad \text{(S4)}$$

Lung tissue

$$C^{Lung} = \frac{\left(Cb^{Lung} \times \left(V_{Vascular}^{Lung} + \left(\frac{V_{Interstitial}^{Lung}}{k(b/p)}\right)\right) + C_{Cellular}^{Lung} \times V_{Cellular}^{Lung}\right)}{V^{Lung}} \quad \text{(S5)}$$

Arterial blood compartment

$$V^{Arterial\ Blood} \times \frac{d}{dt} C^{Arterial\ Blood} = Q^{Lung} \times (Cb^{Lung} - C^{Arterial\ Blood}) \quad \text{(S6)}$$

Spleen compartment

Spleen rapid equilibrium sub-compartment

$$\left(V_{Vascular}^{Spleen} + \left(\frac{V_{Interstitial}^{Spleen}}{k(b/p)}\right)\right) \times \frac{d}{dt} Cb^{Spleen} = Q_{Spleen} \times (C^{Arterial\ Blood} - Cb^{Spleen}) - K_{I\to C}^{Spleen} \times fu \times Cb^{Spleen} \times \left(V_{Vascular}^{Spleen} + \left(\frac{V_{Interstitial}^{Spleen}}{k(b/p)}\right)\right) + K_{C\to I}^{Spleen} \times C_{Cellular}^{Spleen} \times V_{Cellular}^{Spleen} \quad (S7)$$

Spleen cellular sub-compartment

$$V_{Cellular}^{Spleen} \times \frac{d}{dt} C_{Cellular}^{Spleen} = K_{I\to C}^{Spleen} \times fu \times Cb^{Spleen} \times \left(V_{Vascular}^{Spleen} + \left(\frac{V_{Interstitial}^{Spleen}}{k(b/p)}\right)\right) - K_{C\to I}^{Spleen} \times C_{Cellular}^{Spleen} \times V_{Cellular}^{Spleen} \quad (S8)$$

Spleen tissue

$$C^{Spleen} = \frac{\left(Cb^{Spleen} \times \left(V_{Vascular}^{Spleen} + \left(\frac{V_{Interstitial}^{Spleen}}{k(b/p)}\right)\right) + C_{Cellular}^{Spleen} \times V_{Cellular}^{Spleen}\right)}{V^{Spleen}} \quad (S9)$$

Liver compartment

Liver rapid equilibrium sub-compartment

$$\left(V_{Vascular}^{Liver} + \left(\frac{V_{Interstitial}^{Liver}}{k(b/p)}\right)\right) \times \frac{d}{dt} Cb^{Liver} = Q_{Liver} \times (C^{Arterial\ Blood} - Cb^{Liver}) + Q_{Spleen} \times Cb^{Spleen} - K_{I\to C}^{Liver} \times fu \times Cb^{Liver} \times \left(V_{Vascular}^{Liver} + \left(\frac{V_{Interstitial}^{Liver}}{k(b/p)}\right)\right) + K_{C\to I}^{Liver} \times C_{Cellular}^{Liver} \times V_{Cellular}^{Liver} \quad (S10)$$

Liver cellular sub-compartment

$$V_{Cellular}^{Liver} \times \frac{d}{dt} C_{Cellular}^{Liver} = K_{I\to C}^{Liver} \times fu \times Cb^{Liver} \times \left(V_{Vascular}^{Liver} + \left(\frac{V_{Interstitial}^{Liver}}{k(b/p)}\right)\right) - K_{C\to I}^{Liver} \times C_{Cellular}^{Liver} \times V_{Cellular}^{Liver} \quad (S11)$$

Liver tissue

$$C^{Liver} = \frac{\left(Cb^{Liver} \times \left(V_{Vascular}^{Liver} + \left(\frac{V_{Interstitial}^{Liver}}{k(b/p)}\right)\right) + C_{Cellular}^{Liver} \times V_{Cellular}^{Liver}\right)}{V^{Liver}} \tag{S12}$$

Kidney compartment

Kidney rapid equilibrium sub-compartment

$$\left(V_{Vascular}^{Kidney} + \left(\frac{V_{Interstitial}^{Kidney}}{k(b/p)}\right)\right) \times \frac{d}{dt} Cb^{Kidney} = Q_{Kidney} \times (C^{Arterial\ Blood} - Cb^{Kidney}) - K_{I \to C}^{Kidney} \times fu \times Cb^{Kidney} \times \left(V_{Vascular}^{Kidney} + \left(\frac{V_{Interstitial}^{Kidney}}{k(b/p)}\right)\right) - GFR \times fu \times Cb^{Kidney} + K_{C \to I}^{Kidney} \times C_{Cellular}^{Kidney} \times V_{Cellular}^{Kidney} \tag{S13}$$

Kidney cellular sub-compartment

$$V_{Cellular}^{Kidney} \times \frac{d}{dt} C_{Cellular}^{Kidney} = K_{I \to C}^{Kidney} \times fu \times Cb^{Kidney} \times \left(V_{Vascular}^{Kidney} + \left(\frac{V_{Interstitial}^{Kidney}}{k(b/p)}\right)\right) - K_{C \to I}^{Kidney} \times C_{Cellular}^{Kidney} \times V_{Cellular}^{Kidney} \tag{S14}$$

Kidney tissue

$$C^{Kidney} = \frac{\left(Cb^{Kidney} \times \left(V_{Vascular}^{Kidney} + \left(\frac{V_{Interstitial}^{Kidney}}{k\left(\frac{b}{p}\right)}\right)\right) + C_{Cellular}^{Kidney} \times V_{Cellular}^{Kidney}\right)}{V^{Kidney}} \tag{S15}$$

Other tissue compartment

Other tissue rapid equilibrium sub-compartment

$$\left(V_{Vascular}^{Other} + \left(\frac{V_{Interstitial}^{Other}}{k(b/p)}\right)\right) \times \frac{d}{dt} Cb^{Other} = Q_{Other} \times (C^{Arterial\ Blood} - Cb^{Other}) - K_{I \to C}^{Other} \times fu \times Cb^{Other} \times \left(V_{Vascular}^{Other} + \left(\frac{V_{Interstitial}^{Other}}{k(b/p)}\right)\right) + K_{C \to I}^{Other} \times C_{Cellular}^{Other} \times V_{Cellular}^{Other} \tag{S16}$$

Other tissue cellular sub-compartment

$$V_{Cellular}^{Other} \times \frac{d}{dt} C_{Cellular}^{Other} = K_{I \to C}^{Other} \times fu \times Cb^{Other} \times \left(V_{Vascular}^{Other} + \left(\frac{V_{Interstitial}^{Other}}{k(b/p)} \right) \right) - K_{C \to I}^{Other} \times C_{Cellular}^{Other} \times V_{Cellular}^{Other} \tag{S17}$$

Other tissue

$$C^{Other} = \frac{\left(Cb^{Other} \times \left(V_{Vascular}^{Other} + \left(\frac{V_{Interstitial}^{Other}}{k(b/p)} \right) \right) + C_{Cellular}^{Other} \times V_{Cellular}^{Other} \right)}{V^{Other}} \tag{S18}$$

Subcutaneous Dosing

$$V_{Depot}^{Subcutaneous} = 50\ \mu L \tag{S19}$$

$$C_{Depot}^{Subcutaneous} = \frac{F \times Dose}{V_{Depot}^{Subcutaneous}} \tag{S20}$$

$$V_{Depot}^{Subcutaneous} \times \frac{d}{dt} C_{Depot}^{Subcutaneous} = -Ka \times C_{Depot}^{Subcutaneous} \tag{S21}$$

Intra-Pulmonary Aerosol (IPA) Dosing

Dosing depot

$$V_{Depot}^{IPA} \times \frac{d}{dt} C_{Depot}^{IPA} = -Ka \times C_{Depot}^{IPA} \tag{S22}$$

ELF sub-compartment

$$V^{ELF} \times \frac{d}{dt} C^{ELF} = Ka \times C_{Depot}^{IPA} \times V_{Depot}^{IPA} - K_{ELF \to C} \times fu^{ELF} \times C^{ELF} \times V^{ELF} + K_{C \to I}^{Lung} \times C_{Cellular}^{Lung} \times V_{Cellular}^{Lung} - K_{ELF \to B} \times fu^{ELF} \times C^{ELF} \times V^{ELF} + K_{ELF \to B} \times fu \times Cb^{Lung} \times \left(V_{Vascular}^{Lung} + \left(\frac{V_{Interstitial}^{Lung}}{k(b/p)} \right) \right) \tag{S23}$$

Modified lung cellular sub-compartment

$$V_{Cellular}^{Lung} \times \frac{d}{dt} C_{Cellular}^{Lung} = K_{I \to C}^{Lung} \times fu \times Cb^{Lung} \times \left(V_{Vascular}^{Lung} + \left(\frac{V_{Interstitial}^{Lung}}{k(b/p)} \right) \right) - K_{C \to I}^{Lung} \times C_{Cellular}^{Lung} \times V_{Cellular}^{Lung} + K_{ELF \to C} \times fu^{ELF} \times C^{ELF} \times V^{ELF} \tag{S24}$$

Modified lung rapid equilibrium sub-compartment

$$\left(V_{Vascular}^{Lung} + \left(\frac{V_{Interstitial}^{Lung}}{k(b/p)}\right)\right) \times \frac{d}{dt} Cb^{Lung} = Q_{Lung} \times (C^{Venous\ Blood} - Cb^{Lung}) - K_{I\rightarrow C}^{Lung} \times fu \times Cb^{Lung} \times \left(V_{Vascular}^{Lung} + \left(\frac{V_{Interstitial}^{Lung}}{k(b/p)}\right)\right) + K_{C\rightarrow I}^{Lung} \times C_{Cellular}^{Lung} \times V_{Cellular}^{Lung} + K_{ELF\rightarrow B} \times fu^{ELF} \times C^{ELF} \times V^{ELF} - K_{ELF\rightarrow B} \times fu \times Cb^{Lung} \times \left(V_{Vascular}^{Lung} + \left(\frac{V_{Interstitial}^{Lung}}{k(b/p)}\right)\right) \quad \text{(S25)}$$

Modified lung tissue

$$C^{Lung} = \frac{\left(Cb^{Lung} \times \left(V_{Vascular}^{Lung} + \left(\frac{V_{Interstitial}^{Lung}}{k(b/p)}\right)\right) + C_{Cellular}^{Lung} \times V_{Cellular}^{Lung} + C^{ELF} \times V^{ELF}\right)}{V^{Lung}} \quad \text{(S26)}$$

Table S1. Model input parameters for the model development of spectinamide 1599 within the Simcyp simulator.

Parameters	Values
MW (g/mol)	486.95
Log $P_{O:W}$	-2.52
pKa	8.69, 6.95
fu_{Plasma}	0.436
k(b/p)	0.693
CL_R (L/h)	3.27
P_{eff} (10^{-4} cm/s)<br>• Mass to ISF<br>• Blood to ISF<br>• Rim-mass to ISF<br>• OC to ISF<br>• IC to OC	13.89

MW represents molecular weight (g/mol); Log $P_{O:W}$ represents lipophilicity; pKa represents acid dissociation constant; *k(b/p)* represents blood to plasma ratio; and fu_{Plasma} represents fraction unbound in plasma; CL_R is renal clearance; P_{eff} is effective permeability, ISF is interstitial fluid, OC is outer caseum; IC is inner caseum.
